# From Waste to Worth: Innovative Pyrolysis of Textile Waste into Microporous Carbons for Enhanced Environmental Sustainability

**DOI:** 10.3390/polym17030341

**Published:** 2025-01-26

**Authors:** Anastasia Anceschi, Francesco Trotta, Marina Zoccola, Fabrizio Caldera, Giuliana Magnacca, Alessia Patrucco

**Affiliations:** 1 CNR-STIIMA, Italian National Research Council, Institute of Intelligent Industrial Technologies and Systems for Advanced Manufacturing, Corso G. Pella 16, 13900 Biella, Italy; marina.zoccola@stiima.cnr.it (M.Z.); alessia.patrucco@stiima.cnr.it (A.P.); 2 Department of Chemistry, University of Turin, Via P. Giuria 7, 10125 Turin, Italy; francesco.trotta@unito.it (F.T.); fabrizio.caldera@unito.it (F.C.); giuliana.magnacca@unito.it (G.M.)

**Keywords:** recycling, activated carbon, polyester valorization, PET-PU, pyrolysis, thermal conversion

## Abstract

The generation of synthetic textile waste is a growing global concern, with an unsustainable rate of expansion. This study addresses the growing issue of synthetic textile waste by converting polyester–polyurethane (PET-PU) post-industrial scraps into microporous carbon materials, which can be utilized for wastewater treatment. Using a straightforward pyrolysis process, we achieved a high specific surface area (632 m^2^/g) and narrow porosity range (2–10 Å) without requiring chemical activation. The produced carbon materials effectively adsorbed methylene blue and orange II dyes, with maximum adsorption capacities of 169.49 mg/g and 147.56 mg/g, respectively. Kinetic studies demonstrated that adsorption followed a pseudo-second-order model, indicating strong interactions between the adsorbent and dyes. Regeneration tests showed that the C-PET-PU could be reused for multiple cycles with over 85% retention of its original adsorption capacity. Preliminary life cycle assessment (LCA) and life cycle cost (LCC) analysis highlighted the environmental and economic advantages of this upcycling approach, showing a reduced global warming potential and a production cost of approximately 1.65 EUR/kg. These findings suggest that transforming PET-PU waste into valuable adsorbents provides a sustainable solution for the circular economy and highlights the potential for broader applications in environmental remediation.

## 1. Introduction

The shift toward a circular economy represents a top priority for the European Union. This change focuses on the reuse, renewal, and recycling of products and materials, giving new attention to waste as a resource [1,2]. Accordingly, it is increasingly imperative to consider waste as a strategic opportunity to create innovative new products.

The textile industry is one of the most important manufacturing industries, but at the same time, it also has a significant negative impact on the environment due to manufacturing processes and waste production [3]. The increase in textile consumption due to the predominance of fast fashion and push manufacturing has led to a rise in pollutant discharge and waste production [4]. From an environmental standpoint, textile waste can be categorized based on its origin into two main groups requiring two distinct waste management approaches. On one hand, there are post-consumer waste products that result from the disposal of used finished goods. On the other hand, there is post-industrial waste, which includes all by-products generated during the textile production process [5]. Several strategies have been proposed for the management of waste, but particular attention should be paid to post-industrial waste. Post-industrial waste comprises all materials generated during textile manufacturing, including fibers, yarn, fabric, and garment production along the supply chain [5]. Specifically, in the apparel industry, the majority of the waste is fabric scraps, which are rarely reusable [6]. Currently, the fabric scraps are generally landfilled since the dimensions and the composition make them useless for subsequent processing.

To address the environmental impacts of textile waste, there is a growing interest in developing innovative recycling and upcycling methods that transform waste into valuable products. One promising approach involves converting waste into high-performance porous carbon materials for use in environmental remediation applications, such as wastewater treatment. The production of porous carbon from waste materials not only helps mitigate waste accumulation but also provides an economical and sustainable alternative to traditional adsorbents. Multiple research studies focused on exploring the potential of plastic waste as adsorbents, specifically polyester (PET) waste, due to its high availability and elevated carbon content [7]. The transformation of PET waste into carbonaceous material has been extensively studied [8]. Typically, PET is converted into carbonaceous material by thermal treatment. More specifically, PET is pyrolyzed to high temperature under an inert atmosphere in order to convert it into char. In the majority of cases, simple pyrolysis results in the production of carbonaceous material with an extremely low specific surface area and negligible porosity. Consequently, physical or chemical activation procedures must be employed to achieve the desired features [7]. Thus, to enhance the properties of PET-derived carbons, several activating agents have been used, such as CO_2_, alkaline agents, acidic chemicals, and salts [9,10,11,12,13]. In addition, another important factor that generally affects the properties of the carbonaceous material is the structure of the starting materials. For this reason, PET from textiles is not commonly used for its conversion to char, as PET can be found in different forms and shapes. Yuan and colleagues were the first to achieve positive results using PET in the form of fibers, but in combination with magnesium salts as an activating agent [7]. To complicate matters, in addition to the structure of the starting materials, in the textile sector, PET is usually blended with other polymers to achieve desired properties in the final product [14]. For example, to improve the elastic properties of the final products, PET is often blended with polyurethane (PU), commonly called elastane or spandex. In general, the co-pyrolysis of PET with other polymers affects the short-chain aromatic intermediates produced during pyrolysis, reducing the quantity and quality of carbon produced [15]. Therefore, polymer blends are generally not considered as precursors for carbon materials.

Despite these concerns, this study examined the production of carbon materials from PET blended with polyurethane. While the production of carbon materials from polymer waste can have beneficial environmental and economic outcomes, it would be crucial to ascertain and evaluate the financial implications and the overall impact of the entire process. To date, no comprehensive study that couples in-depth laboratory production of carbon material from textile waste with an environmental impact assessment has been conducted. Given the global concern about the environmental impact of new processes and the importance of production costs in this context, it is crucial to identify and analyze the parameters associated with the production of carbonaceous materials from textile waste. One simple and practical methodology for assessing the potential environmental impact of a production process is the life cycle assessment (LCA). The objective of the LCA analysis is to investigate the environmental impacts of processing activities in order to comply with several requirements, such as quality and performance, technical feasibility, cost control, and respect for the environment [16]. The analysis plans to identify viable solutions to enhance the environmental performance of a production system. Nevertheless, in the context of a potential large-scale production of carbon materials, it is essential to consider not only the LCA analysis but also the life cycle cost (LCC) assessment. The LCC is a powerful tool that enables the determination of the cost-efficiency of a production process [17].

This study investigates the feasibility of converting PET-PU post-industrial scraps into microporous carbon materials through pyrolysis. We aimed to evaluate the adsorption performance of these materials for dye removal, focusing on methylene blue and orange II as model contaminants. Additionally, we conducted regeneration and reusability studies to assess the long-term viability of the adsorbents. Finally, we performed a comprehensive life cycle assessment (LCA) and life cycle cost (LCC) analysis to quantify the environmental and economic benefits of this upcycling process. Our findings demonstrate the potential of using PET-PU waste as a sustainable feedstock for high-value carbon materials, contributing to the advancement of circular economy practices in the textile industry.

## 2. Materials and Methods

### 2.1. Materials

The PET-PU warp-knitted fabric scraps (PET-PU) were kindly provided by Radici Group (Gandino, Italy). The composition is 87% of PET and 13% of PU. Methylene Blue (MB) and Orange II (OII) were purchased from Sigma Aldrich (Darmstadt, Germany).

### 2.2. Carbon Preparation

The PET-PUs were cut and transferred to an alumina pan and then heated to 800 °C with a temperature ramp of 10 °C min^−1^ in a nitrogen gas flow (65 mL min^−1^). The apparatus was allowed to settle under the nitrogen flux until room temperature was reached.

### 2.3. Morphological Characterization

A morphology examination was performed using a Zeiss (Oberkochen, Germany) EVO 10 scanning electron microscope on the PET-PU warp-knitted fabric scraps and the resulting C-PET-PU carbon material. The following parameters were selected: 15–20 kV acceleration voltage and 50 pA current probe. The samples were fixed on aluminum stubs and deposited with gold in rare argon using an Emitech (Corato, Ba, Italy) K 550 sputter coater at a current of 20 mA for 180 s.

### 2.4. Thermal Analysis

Thermogravimetric analysis was performed in a TGA Mettler Toledo (Milan, Italy), model Stare system. The system worked under a nitrogen atmosphere (gas flow: 100 mL min^−1^). Ramp heating from room temperature to 800 °C at 10 °C min^−1^ was carried out on approximately 10 mg of sample mass in an open alumina pan.

### 2.5. Fourier Transformed Infrared Spectroscopy (FTIR)

The FTIR spectra of materials were acquired with the Attenuated Total Reflection (ATR) mode in the range of 4000 to 650 cm^−1^ with 64 scans and a resolution of 4 cm^−1^ with a Thermo Nicolet (Waltham, MA, USA) Nexus spectrometer with ZnSe crystal.

### 2.6. Gas-Volumetric Analysis

The carbons obtained after the pyrolysis process were characterized using an automated adsorption apparatus (ASAP 2020, Micromeritics, Norcross, GA, USA) to obtain nitrogen adsorption–desorption isotherms. The samples were outgassed overnight at 300 °C and analyzed with nitrogen at 77 K. The Langmuir model was used to evaluate the specific surface area, while the Density Functional Theory (DFT) model was used to determine the pore number and dimension, considering a pore slit geometry.

### 2.7. Pyrolysis Kinetic Analysis

Samples were heated from room temperature to 800 °C at different heating rates (5, 10, 15, and 20 °C/min) under a nitrogen atmosphere with a flow rate of 100 mL/min. Then, the Kissinger–Akahira–Sunose (KAS) and Flynn–Wall–Ozawa (FWO) methods were used to determine the activation energy (E_a_) of the pyrolysis process. In particular, the KAS method is based on the following equation [18]:

(1) $$ln\left(\frac{\beta }{T^{2}}\right)=ln\left(\frac{AR}{E_{a}}\right)-\frac{E_{a}}{RT}$$

where b is the heating rate, T is the absolute temperature, A is the pre-exponential factor, R is the gas constant, and E_a_ is the activation energy.

For the FWO method, the equation is as follows [19]:

(2) $$log\left(\beta \right)=log\left(\frac{{AE}_{a}}{R}\right)-2.315-\frac{{0.4567E}_{a}}{RT}$$

By plotting log(β) against 1/T for each conversion value (α), the activation energy Ea can be obtained from the slope of the linear fit.

### 2.8. Simulative Analysis of the Structure of C-PET-PU

To better understand the carbon structure, a simulative analysis was performed using the Large-scale Atomic/Molecular Massively Parallel Simulator (LAMMPS). The setup utilized Adaptive Intermolecular Reactive Empirical Bond Order (AIREBO) potential for carbon atoms. For the initial structure, a representative model of PET-PU was constructed based on experimental composition (87% PET, 13% PU). The details of the simulation procedure are as follows:

1. Construction of PET-PU Model: A polymeric model of PET-PU was built with a random distribution of PET and PU segments.
2. Equilibration: The model was equilibrated at 300 K for 500 ps to relax the structure.
3. Pyrolysis Simulation: The equilibrated structure was gradually heated to 800 °C at a rate of 10 °C/min under an inert nitrogen atmosphere. The simulation was carried out for 1 ns.
4. Cooling and Annealing: The system was cooled to room temperature under a continuous nitrogen flow and annealed to stabilize the carbon structure.
5. Analysis: The final structure was analyzed for atomic arrangement, pore distribution, and surface functionalities.

### 2.9. Dyes Used in the Study

In this study, methylene blue (MB) and orange II (OII) were selected as model dyes to evaluate the adsorption performance of the C-PET-PU materials. These dyes are representative of common contaminants in industrial effluents due to their widespread use in textiles, printing, and other industries, as well as their distinct chemical and physical properties.

Methylene Blue (MB) is a cationic dye with a molecular formula of C_16_H_18_ClN_3_S and a molar mass of 319.85 g/mol. MB is characterized by its high solubility in water, its intense blue color, and its toxicity to aquatic life in high concentrations [20]. Its adsorption on negatively charged surfaces provides insight into electrostatic interaction mechanisms. In contrast, OII is an anionic dye with a molecular formula of C_16_H_11_N_2_NaO_4_S and a molar mass of 350.32 g/mol. It is classified as an azo dye and is prevalent in the dyeing of wool, silk, and synthetic fibers [21]. Its persistence and potential to degrade into toxic aromatic amines under environmental conditions make it a critical pollutant.

The selection of both dyes was based on their contrasting charge properties, thereby enabling a comprehensive evaluation of the C-PET-PU’s adsorption mechanisms and effectiveness. Moreover, the utilization of these dyes mirrors real-world scenarios where effluents may contain both cationic and anionic dyes.

### 2.10. Adsorption Evaluation

Different dye solutions ranging from 1 to 50 ppm of MB or OII were prepared. The carbon obtained from PET-PU waste was immersed in a dye solution at a 1:50 liquor ratio. The mixture was continuously stirred while varying the contact time from 30 s to 48 h. The resulting solutions were filtered through a PTFE syringe filter with a pore width of 0.45 µm. The adsorption was assessed using a Perkin Elmer 200 double bean UV-Vis spectrophotometer at 650 nm for MB and 486 nm for OII.

The following equation was used to quantify the amount of dye adsorbed at equilibrium (qe, mg g^−1^):

(3) $$q_{e}=\frac{V\left(C_{0}-C_{e}\right)}{m}$$

The volume of the solution tested was 10 mL (V), while the initial dye concentration in solution (C_0_) and the dye concentration at equilibrium (C_e_) were expressed in mg L^−1^. The amount of sorbent used was 10 mg (m).

The equilibrium experimental data were analyzed in accordance with the Langmuir and Freundlich models. The Langmuir model was applied using the formula below:

(4) $$\frac{C_{e}}{q_{e}}=\frac{C_{e}}{q_{m}}+\frac{1}{K_{L}q_{m}}$$

C_e_ and q_e_ represent the steady-state dye concentration (mg L^−1^) and the quantity of adsorbed (mg), respectively. q_m_ and K_L_ represent the Langmuir constants that correspond to the adsorption capability (mg g^−1^) and the adsorption steady-state constant (L g^−1^), respectively.

(5) $$logq_{e}=logK_{F}+(1/n)logC_{e}$$

K_F_ and n are Freundlich constants related to adsorption capacity and intensity, respectively.

In this study, adsorption data were evaluated using two kinetic models, pseudo-first-order and pseudo-second-order. The pseudo-first-order model is described by [22]:

(6) $$log{(q}_{e}-q_{t})=logq_{e}-(K_{1}/2.303)t$$

The amounts of dye (mg g^−1^) adsorbed on the PET-PU-derived carbon at steady state and at time t are represented by q_e_ and q_t_, respectively. The constant rate (min^−1^) is denoted by k_1_. To obtain the value of k_1_, the slope of the linear plots of log(q_e_ − q_t_) vs. t was calculated.

The data were fitted to the pseudo-second-order mechanism as follows [23]:

(7) $$t/q_{t}=(1/h)+(1/q_{e})t$$

The initial rate of adsorption h is as follows:

(8) $$h=k_{2}q_{e}^{2}$$

The validity of the pseudo-second-order kinetic model is assured by the linear fit of the plot of t/q_t_ versus t (Equation (6)), from which the constants q_e_, h, and k_2_ can be derived. k_2_ is the constant rate of pseudo-second-order adsorption (g mg^−1^ min^−1^), and h is the initial adsorption rate (mg g^−1^ min^−1^).

Thermodynamic of the dyes adsorption, enthalpy, ∆H^0^, entropy, ∆S^0^, and free energy of adsorption, ∆G^0^ are computed as follows [24]:

(9) $$K_{c}=\frac{C_{Ae}}{C_{e}}$$

(10) $${\Delta G}^{0}=-RTlnK_{c}$$

(11) $${\Delta G}^{0}=\Delta H^{0}-T\Delta S^{0}$$

### 2.11. Regeneration and Reusability Studies of C-PET-PU

The regeneration and reusability of the C-PET-PU were investigated to evaluate their potential for practical application. In this instance, the adsorption and desorption studies were conducted with a particular focus on the use of MB. Specifically, 100 mg of C-PET-PU was introduced to a 100 mL solution of MB with a concentration of 50 mg/L. Subsequently, the mixture was stirred at room temperature for 24 h, after which the adsorbent was separated by filtration. The concentration of the dye in the solution was then measured using a UV-Vis spectrophotometer (PerkinElmer, Turin, Italy) at 650 nm for the MB. Subsequent to the completion of the adsorption process, the C-PET-PU was soaked for a period of two hours in a solution consisting of 100 mL of ethanol to desorb the adsorbed MB. Thereafter, a further washing step was conducted using distilled water, followed by a drying process at 80 °C for a period of four hours. Subsequently, the regenerated C-PET-PU was subjected to another adsorption cycle under identical conditions, and this process was repeated five times to assess the reusability of the material. To calculate the regeneration efficiency (REn), the following formula has been used [25]:

(12) $$R_{n}=\left(\frac{q_{e,n}}{q_{e,0}}\right)\times 100$$

where qe,0 is the initial adsorption capacity, and q_e,n_ is the adsorption capacity after n cycles.

### 2.12. LCA Analysis

For the LCA analysis, the software Umberto academic version (Ipoint-system GmbH Reutlingen, Germany )was used with the USLCI database. In this preliminary LCA analysis performed, the results were provided as global warming potential indicators. The objective of this study is to evaluate the environmental consequences associated with the transformation of PET-PU waste into microporous carbon. For the assessment, it was estimated that 100 kg of PET-PU post-industrial scrap would be treated in a reactor with a capacity of 100 kg. In this scenario, the treatment of PET-PU was simulated, and the energy requirements, gas production, and nitrogen consumption for treating 100 kg of waste were evaluated. It was thus estimated that the entire process would take 95 min, with the cooling phase occurring naturally. The data pertaining to the energy requirements for pyrolysis, nitrogen consumption, and process yield were obtained through direct measurement. The remaining data were extrapolated from published literature sources and normalized with respect to the specific contribution of the two main components (87% PET and 13% PU) [26,27,28,29]. Finally, this LCA study aims to evaluate waste treatment within the geographical boundaries of Italy.

### 2.13. LCC Analysis

Similar to the LCA, the life cycle cost (LCC) analysis was performed using Umberto as software with the same functional unit (100 kg of treated PET-PU waste). For this purpose, the inventories were integrated with the cost database i.e., raw materials, gas abatement, and energy required in Italy. The LCC performed was based on the pilot-lab-scale process.

## 3. Results

### 3.1. Thermal Analysis

**Figure 1:**
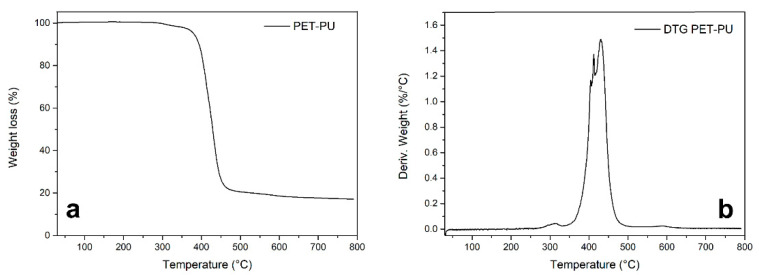
TGA (**a**) and DTG (**b**) curves of PET-PU.

In order to investigate the pyrolysis temperature, the PET-PU was analyzed using the TGA. The TGA result is reported in Figure 1.

Figure 1 displays the TGA (a) and differential thermogravimetric (DTG) curves (b) of PET-PU. According to the DTG, the thermal decomposition profile reveals two steps. The initial decomposition stage led to a 3% weight loss at temperatures ranging from 270 °C to 350 °C. In this stage, the weight loss can be ascribed to the initial degradation of the PU material, which has been demonstrated in previous studies [30]. Significant weight loss (77%) occurred during the second stage of degradation, which occurred between 350 °C and 500 °C. This stage primarily involves the degradation of PET, and in the meantime, macromolecular aromatic compounds have been generated to produce carbonaceous materials by means of radical polymerization reaction [31]. Weight loss remains constant during the 500–750 °C stage, causing no major alterations in the PET-PU mass and yielding 20% of char. This suggests that PET-PU can be transformed into the final product by setting the pyrolysis temperature to 700 °C.

### 3.2. Pyrolysis Kinetic Analysis

To elucidate the kinetic mechanism of the pyrolysis of PET-PU post-industrial waste, TGA and model-free kinetic methods were employed. Specifically, the PET-PU was subjected to a pyrolysis process using the TGA at varying ramping rates, from 5 to 20 °C. Subsequently, the Kissinger–Akahira–Sunose (KAS) and Flynn–Wall–Ozawa (FWO) methods were employed to analyze the TGA data, thereby determining the activation energy (Ea) of the pyrolysis process. The calculated activation energies for the two degradation stages using both KAS and FWO methods are summarized in Table 1.

The activation energies obtained for the PET degradation stage are higher than those for PU degradation, which is indicative of the more stable aromatic structure of PET in comparison to the urethane linkages in PU. The consistency between the KAS and FWO methods serves to validate the reliability of the determined kinetic parameters. The reaction mechanism can be described as involving complex reactions due to the presence of two polymers, namely PET and PU, in the pyrolysis process. Polyurethane decomposes primarily through random scission and depolymerization, resulting in the release of volatile products. The decomposition of PET involves the cleavage of ester bonds, resulting in the formation of char and volatile aromatic compounds. The interaction between PET and PU during pyrolysis has the potential to influence the yield and properties of the resulting carbon material.

### 3.3. Specific Surface Area and Porosity Analysis

**Figure 2:**
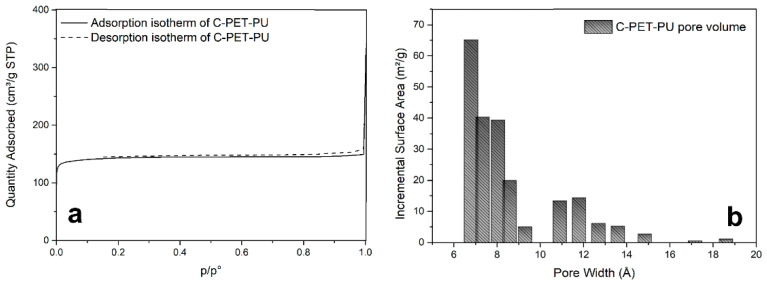
Isotherm plot (**a**) and incremental pore volume (**b**) of C-PET-PU.

The N2 adsorption/desorption isotherm was used to investigate the textural properties of the C-PET-PU. According to the IUPAC classification, the C-PET-PU nitrogen adsorption isotherm at a temperature of −196 °C can be classified as Type I, suggesting that the sample is predominantly a microporous material [32]. The desorption isotherm indicated reversibility, suggesting no development of mesoporosity. The results of the porosity study and the measured isotherm are shown in Figure 2.

As the isotherm in Figure 2a matches the Type I isotherm criteria, the specific surface area has been calculated using the Langmuir equation. Surprisingly, the estimated value of the specific surface area is 632 m^2^/g, a relatively high value compared to the values obtained in the literature [33,34,35,36]. Higher specific surface areas have been obtained with pure PET, but some activating agents must be used. In this case, good results were achieved in PET-PU pyrolysis without any activating agents. According to the DFT method, the pore size distribution (Figure 2b) indicates a pore volume of 0.8 cm^3^/g with a close distribution of micropores ranging from 2–10 Å. These porosity values are interesting because the development of specific surface area and microporosity is typically inhibited by the presence of mixed polymers [37,38,39]. Typically, such blending reduces the carbonization yield and is therefore usually avoided in the production of carbonaceous materials. However, in the case of PET-PU pyrolysis, the structure of the fabric, as well as the presence and arrangement of polyurethane, seem to have a beneficial effect on the specific surface area development and control over porosity, leading to the formation of a microporous carbonaceous material with a relatively high surface area.

### 3.4. Morphology Investigation

**Figure 3:**
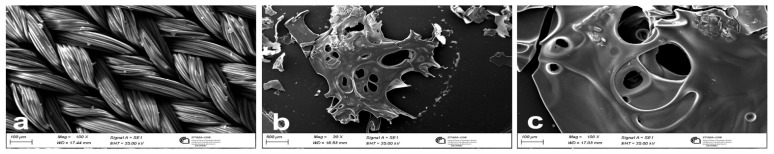
SEM micrographs of (**a**) PET-PU (100×); (**b**) C-PET-PU (20×); (**c**) C-PET-PU (100×).

The morphology of the PET-PU fabric was examined using the SEM microscope before and after pyrolysis, as shown in Figure 3.

Figure 3a displays 100× magnified images of PET-PU fabric. The sample exhibited a typical morphology, possessing a smooth surface with a diameter of 17 mm. Figure 3b,c depict the carbon obtained after pyrolysis of the PET-PU at magnifications of 20× and 100×, respectively. Interestingly, the particles show a highly heterogeneous morphology. The explanation is that as the temperature increases, PET first reaches its melting point and then decomposes. Two competitive reactions occur during PET decomposition: one results in char formation, and the other produces volatile compounds that escape from the melted phase of PET. Concurrently with the degradation of PET, the PU also undergoes decomposition, leading to the release of a significant quantity of volatile products. The series of reactions outlined during the PET-PU decomposition culminates in the formation of the ultimate carbonaceous porous structure.

### 3.5. FT-IR Analysis

**Figure 4:**
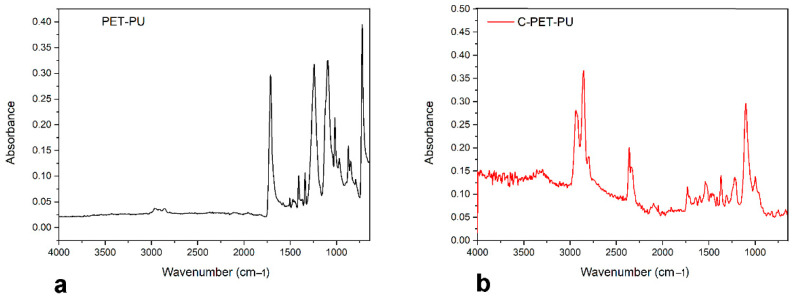
IR spectrum of PET-PU (**a**) and C-PET-PU (**b**).

To gain insight into the composition and surface functional groups of the sample resulting from the pyrolysis of PET-PU, an FTIR analysis has been performed. The collected spectra of the PET-PU (a) and C-PET-PU (b) are reported in Figure 4.

In the PET-PU spectrum, the typical adsorption band can be observed [40]. As shown in Figure 4a, the peaks at 3100–2800 cm^−1^ are attributed to aromatic and aliphatic –CH bond stretching, whereas the band at 1720 cm^−1^ is ascribable to the ester carbonyl bond stretching, and the bands at 1300 cm^−1^ and 1100 cm^−1^ can be attributed to the C-C-O stretching and to O-C-C stretching, respectively. In the C-PET-PU spectrum shown in Figure 4b, some characteristic functional groups could be noticed. The spectrum reveals the presence of weak peaks attributable to CH stretching vibrations between 3500 and 3250 cm^−1^ [41]. Furthermore, the peak located between 2926 cm^−1^ and 2798 cm^−1^ shows evidence of the presence of a predominantly carbon-based structure produced after the pyrolysis of PET-PU, through the stretching of -CH bonds. Additionally, other functionalities can be identified. The bands at 2363 cm^−1^ and 2368 cm^−1^ indicate the presence of nitrogen-containing groups, while the bands at 1724 cm^−1^ and 1100 cm^−1^ suggest the presence of residual hydroxyl and carboxyl groups. Additionally, a band is observed at 995 cm^−1^, which is related to the sp2 C=C and aromatic ring associated with the charring process of PET-PU [42]. Therefore, it can be assumed that the pyrolysis has converted the PET-PU into a graphitized-like structure but has left some residual functional groups with a hydrophilic nature, which is beneficial for its dispersion and adsorption in aqueous systems.

### 3.6. Simulative Analysis of the Structure of C-PET-PU

The conversion of PET-PU post-industrial waste into carbon materials through pyrolysis gives rise to a complex microstructure, exhibiting a high specific surface area and notable microporosity. To supplement the experimental findings and gain a more comprehensive understanding of the material’s properties, molecular dynamics (MD) simulations were employed. This simulative approach allows for a thorough examination of the atomic configuration, pore distribution, and surface characteristics, which are essential for understanding the material’s potential for adsorption applications. In particular, the molecular dynamics simulation demonstrated that the pyrolysis process results in the formation of an amorphous carbon structure comprising randomly distributed graphitic and disordered carbon regions. The presence of sp^2^-hybridized carbon atoms (graphitic domains) was observed alongside sp^3^-hybridized carbon atoms (disordered regions). This hybrid structure is responsible for the high specific surface area observed in the experimental results. Moreover, the pore size distribution was examined using a geometric approach. The results demonstrate a prevalence of micropores within the range of 2–10 Å, which is consistent with the experimental nitrogen adsorption–desorption isotherms. A comparison between the theoretical and the experimental data is summarized in Table 2.

The total pore volume was calculated to be 0.8 cm^3^/g, which is in alignment with the findings of the experimental investigation. Indeed, the simulated specific surface area (635 m^2^/g) was in close agreement with the experimental value (632 m^2^/g), thus validating the accuracy of the MD model.

### 3.7. Adsorption Evaluation

Since the C-PET-PU has proven to have the required characteristics in terms of porosity, surface morphology, and chemistry to be used as an adsorbent and taking into account the textile-to-textile recycling loop, its ability to adsorb dye probes like the MB and OII was investigated. Firstly, the effect of initial dye concentration has been studied at different MB and OII concentrations (from 10 to 150 ppm), and the obtained results are reported in Table 3.

Generally, the increase in initial concentration is strongly related to the necessary driving force to overcome the resistance to the mass transfer from the liquid phase to the solid phase, and therefore, a sharp rise in the concentration causes an increase in the amount of dye adsorbed. In this case, the quantity of dye adsorbed is independent of the initial dye concentration; hence, the MB and OII concentration gradient did not enhance the dye uptake. Interestingly, the amount of MB that can be absorbed by the C-PET-PU is higher compared to the quantity adsorbed of OII. This difference in the amount of dye adsorbed can be attributed to the presence of certain acidic functionalities, such as carboxylic groups present in the carbon structure. These acidic functionalities enhance the adsorption of cationic species like MB and consequently increase the adsorptive capacity of the C-PET-PU. Therefore, the porous structure and the presence of residual functional groups make the material more selective toward certain species. Nevertheless, it is important to note the potential disparity between the molecular dimensions of the dyes and the pore size of the C-PET-PU material. MB has an approximate molecular size of 13.6 Å × 6.8 Å × 3.2 Å, while OII measures around 12.0 Å × 6.0 Å × 3.0 Å [43]. Given the microporous nature of C-PET-PU, with a pore size distribution predominantly in the range of 2–10 Å, it is plausible that the adsorption process primarily occurs through electrostatic interactions on the external surface or at the pore entrances, rather than within the micropores themselves.

**Figure 5:**
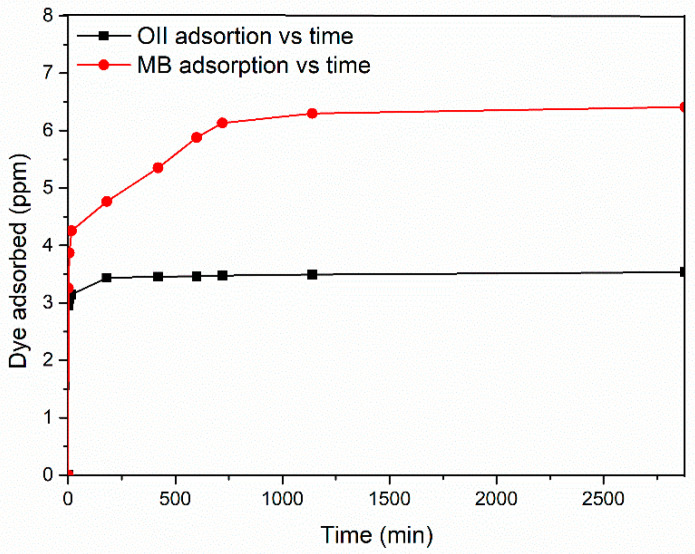
Effect of contact time for OII and MB on C-PET-PU.

Another fundamental parameter related to the mass transfer phenomena, such as adsorption, is the contact time. This parameter represents the time required for the dye-substrate system to achieve equilibrium [44]. Therefore, in order to evaluate the equilibrium time needed to reach the MB and OII maximum adsorption at a fixed concentration, the amount of dyes adsorbed was estimated to vary from 1 to 2880 min, and the results are reported in Figure 5.

The effect of contact time on the removal of OII and MB is shown to be different in Figure 5. In both cases, the availability of active sites on the outer surfaces of the material resulted in a rapid adsorption of the dyes, particularly at the early stages of the adsorption process. Once the active sites were saturated, the dyes entered the pores of the C-PET-PU at a slower rate, eventually reaching equilibrium. Specifically, for OII, dye adsorption exhibits a rapid initial increase over the first 180 min, after which there is a sharp decline, and it reaches a plateau at a concentration of 3.53 ppm. The results indicate that the rate of dye uptake reaches a plateau at the maximum contact time, suggesting the attainment of a state of equilibrium. In the case of MB, the adsorption capacity demonstrates a sharp rise in the initial stages of the experiment, reaching 4.76 ppm after 180 min and 6.1 ppm after 720 min. However, beyond this point, there is no observable improvement in dye adsorption, and a steady state is achieved.

Given that no improvement in adsorption was observed following an increase in contact time beyond 180 min for the OII and 720 min for MB, these times were selected to perform additional thermodynamic experiments.

**Figure 6:**
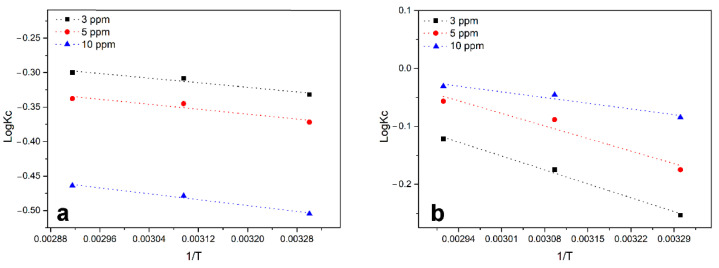
LogKc vs 1/T curves for (**a**) OII adsorption and (**b**) MB adsorption.

For a better understanding of the mechanisms behind the adsorption of the two dyes onto the C-PET-PU, relevant thermodynamic parameters were fully investigated. Specifically, by analyzing the Van’t Hoff plot of logKc versus 1/T at 303 K, 313 K, and 343 K (Figure 6), the relative ∆H^0^ and ∆S^0^ associated with the adsorption of MB and OII were calculated. The obtained ∆H^0^ and ∆S^0^ values were then used to calculate free energy (∆G^0^) related to the adsorption of the two selected dyes.

Thermodynamic parameters offer a comprehensive insight into the inherent energetic changes occurring during adsorption. Therefore, changes in ∆G^0^, ∆H^0^, and ∆S^0^ were calculated to clarify the adsorption process; the results are reported in Table 4.

The Gibbs free energy change ΔG^0^ values were consistently negative across the temperature range tested, indicating that the adsorption of OII and MB onto C-PET-PU was thermodynamically feasible and spontaneous. With regard to OII, as the temperature rises, the ΔG^0^ value becomes increasingly negative, indicating that adsorption is more spontaneous at higher temperatures. In contrast, for MB, as the temperature increases, the ΔG^0^ of the reaction becomes progressively less negative, suggesting a decreasing spontaneity of adsorption at higher temperatures. With regard to enthalpy, both dyes exhibit a negative ΔH^0^ value, thereby indicating that the adsorption process is exothermic in nature. As observed for ΔG^0^, the ΔH^0^ value for MB becomes less negative as the temperature increases. The positive ΔS^0^ values obtained from OII and MB adsorption studies on C-PET-PU indicate that the degree of freedom at the solid–liquid interface is enhanced during the adsorption process. The effectiveness of dye removal has been demonstrated for materials with a high surface area and microporosity, such as the C-PET-PU. [45]. Nevertheless, in the case of C-PET-PU, the results indicate weak correlations between the removal capacities of dye and the micropore volume. Therefore, the influence of the chemical composition of the sorbent on the dye uptake cannot be completely excluded in this study. Taking this into account, the differences in the OII and MB adsorption process can be correlated with a different interaction of each dye with the carbon surface. The driving force of adsorption can reasonably be attributed to the electrostatic interaction between the charged surface of C-PET-PU and the dye molecules. Since the FTIR analysis shows that there are some residual carboxylic and hydroxylic groups on the surface of C-PET-PU, it is reasonable to assume that the surface is negatively charged. As the surface of C-PET-PU is slightly negative, it could electrostatically attract positively charged molecules such as MB. By increasing the temperature, thermal agitation generates a reduction in electrostatic attraction, making the adsorption process less preferred. As far as OII is concerned, it may also have some interaction with some functional residues present on the surface in a minor way. Indeed, a recent study has shown that the presence of some specific functional groups on both the adsorbent and the adsorbate determines the affinity of sorbents for a surface. In particular, nitrogen-containing substrates have a high affinity for anionic dyes with sulfonate groups [46].

**Figure 7:**
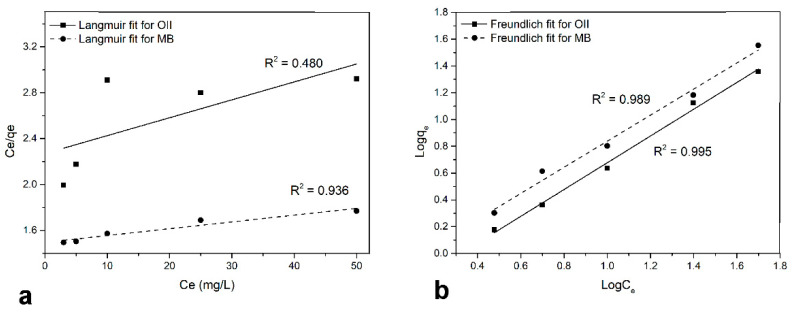
(**a**) Langmuir and (**b**) Freundlich isotherm plots for adsorption of OII (solid line) and MB (dotted line) onto C-PET-PU.

The adsorption isotherms of MB and OII were calculated to determine the adsorption capacity of C-PET-PU. The equilibrium data were fitted to the Langmuir and Freundlich Equations (4) and (5). The Langmuir equation assumes that the highest adsorption corresponds to the saturated monolayer of solute molecules on the surface of C-PET-PU and that the adsorption energy is constant without the interaction of adsorbed molecules on the surface. Adsorption on heterogeneous surfaces with interacting adsorbate molecules is described by the Freundlich isotherm model. The Langmuir (a) and Freundlich (b) isotherms for both dyes are shown in Figure 7.

The equilibrium data for the adsorption of MB are consistent with the Langmuir isotherm, with a correlation coefficient of R^2^ = 0.936, as shown in Figure 7a. These findings indicate that the adsorption of OII and MB on the surface of C-PET-PU occurs via distinct adsorption pathways. Indeed, the preferential adsorption of MB molecules to specific sites on the surface of the material leads to saturation coverage, resulting in a full occupation of these sites. In contrast, neighboring adsorbed molecules are unable to interact with each other. At equilibrium, the number of MB molecules adsorbed is equal to the number of molecules desorbed, and the rate of adsorption and desorption is balanced. The Langmuir monolayer adsorption capacity (qm) was calculated at 169.49 mg g^−1^, with a KL value of 3.94 × 10^−3^, indicating a significant MB–carbon surface interaction. Interestingly, as shown by the value of the coefficient of determination (R^2^ = 0.480), the Langmuir fit is not applicable to the adsorption of OII on C-PET-PU. Since Langmuir cannot be used to model OII adsorption, it can be inferred that adsorption is not completely monolayer-saturation-dependent. As shown in Figure 7b, the equilibrium data for OII and MB adsorption are consistent with the Freundlich equation. This model indicates that adsorption energy declines exponentially as the available sites on the surface of C-PET-PU are fully occupied. Therefore, the Freundlich constants KF and n were evaluated in order to define the multilayer adsorption capacity for both dyes. The KF value was found to be 0.323 for the adsorption of OII and 0.136 for the adsorption of MB. The heterogeneity of the adsorbent was investigated and its correlation with the adsorption intensity was assessed by calculating n values. A value of n ≪ 1 indicates that the adsorption intensity is adequate for the whole range of concentrations studied. If the estimated value of n is n > 1, the adsorption intensity progresses at high concentrations. For both OII and MB, the n values obtained during adsorption were approximately 1.00, implying that an increase in concentration slightly enhances the adsorption. These outcomes indicate that the adsorption of OII and MB onto the surface of the C-PET-PU is principally a physical process.

### 3.8. Kinetic Studies

The adsorption process mechanism was studied from a kinetic point of view by applying the pseudo-first-order and pseudo-second-order models to OII and MB adsorption. Table 5 lists the results of the rate constant studies obtained by the pseudo-first-order and pseudo-second-order models.

According to Table 5, the obtained data for the adsorption of OII and MB indicate poor fitting with the pseudo-first-order model, as evidenced by the low correlation coefficients R^2^ obtained (0.782 and 0.753 for OII and MB, respectively). Nevertheless, the high R^2^ value (>99%) of the pseudo-second-order model suggests that this model more accurately describes both OII and MB adsorption.

Despite the high R^2^ value recorded for OII (99.9%), the significant difference between the experimental q_e_ (3.53 mg/g) and the calculated q_e_ (0.55 mg/g) indicates that the pseudo-second-order model is an inadequate model for the experimental data. In contrast, the pseudo-second-order plots for MB demonstrate an excellent fit with a high R^2^ value (99.7%), and the calculated q_e_ (6.90 mg/g) is in close agreement with the experimental q_e_ (6.76 mg/g). Therefore, the adsorption process can be accurately explained by the pseudo-second-order model solely for MB. Furthermore, the pseudo-second-order kinetic model provides evidence that chemical interactions occurring at the adsorbent surface functional group interface with the MB molecules may be the dominant factor governing the rate of adsorption.

### 3.9. Comparison with Literature Data

The adsorption performance of C-PET-PU for MB and OII dyes was evaluated and compared with similar studies using carbonaceous adsorbents derived from different materials. Table 6 summarizes the adsorption capacities reported in this study alongside data from the literature for comparable adsorbents.

As illustrated in Table 6, the adsorption capacity of C-PET-PU for methylene blue (169.49 mg/g) is notable when compared to that of various adsorbents derived from both renewable and non-renewable feedstocks. It is evident that the adsorption capacity of C-PET-PU for MB (169.49 mg/g) is competitive with several conventional adsorbents, including those derived from citrus fruit peel (25.51 mg/g) and sunflower oil cake (15.80 mg/g). While pure PET-derived carbons exhibit higher adsorption capacities, they require chemical activation, which adds to production costs and environmental impacts. In contrast, C-PET-PU achieves a balance between performance and sustainability by utilizing post-industrial PET-PU waste without activation to produce high-performance carbon materials. This competitive performance underscores the potential of C-PET-PU as a cost-effective and environmentally friendly adsorbent for dye removal in wastewater treatment applications.

### 3.10. Regeneration and Reusability Studies of C-PET-PU

The regeneration and reusability of adsorbent materials are critical for assessing their practical applicability and cost-effectiveness in real-world applications. Therefore, the regeneration efficiency and reusability of C-PET-PU over multiple adsorption–desorption cycles were evaluated, focusing on its performance for methylene blue dye adsorption. The obtained results are listed in Table 7.

The results demonstrated that C-PET-PU maintained a high regeneration efficiency, retaining over 85% of its original adsorption capacity after five consecutive adsorption–desorption cycles. This remarkable performance can be attributed to the robust structural integrity and chemical stability of the carbon framework, which withstands repeated regeneration without significant degradation. The use of ethanol as a desorption agent effectively removed adsorbed MB molecules, indicating that the adsorption mechanism is primarily physical, involving electrostatic attractions between the dye molecules and the carbon surface. Therefore, the retention of adsorption capacity over multiple cycles highlights the potential for C-PET-PU to be used as a cost-effective and sustainable adsorbent in wastewater treatment processes. The minimal loss in adsorption performance suggests that C-PET-PU can effectively compete with conventional adsorbents, such as activated carbon, in applications where economic and environmental considerations are paramount. The high regeneration efficiency of C-PET-PU translates into significant economic benefits, as it reduces the frequency of adsorbent replacement and minimizes the operational costs associated with continuous dye removal processes. The ability to regenerate and reuse the adsorbent multiple times extends its lifecycle, thereby decreasing the overall cost per use and enhancing its attractiveness for large-scale industrial applications. Environmentally, the regeneration process contributes to the sustainability of the adsorption system by lowering the environmental footprint associated with the production and disposal of adsorbents. The use of ethanol, a relatively benign solvent, further reduces the potential environmental impact compared to harsher chemical regeneration methods. Additionally, the circular economy approach of converting waste materials into valuable products aligns with global efforts to reduce waste and promote sustainable resource management.

### 3.11. LCA and LCC Analysis on Conversion of PET-PU into Carbon Material

In order to assess the sustainability of the conversion of textile waste into carbon materials, a preliminary LCA and LCC analysis has been conducted. Firstly, the primary data obtained from laboratory data collection and literature were utilized within LCI. In the event that data were unavailable from the laboratory analysis, the following papers were consulted in order to supplement the missing data [26,27]. For example, with regard to the gas developed during pyrolysis, it was assumed for simplicity that the PET and PU did not interact during the pyrolysis process. The typology and quantity of major gases generated during the pyrolysis of PET and PU are presented in Table 8.

After consideration of the emissions, only the predominant components were calculated, summed, and normalized according to the percentage in weight-to-weight (*w*/*w*) of PET and PU within the blend. Thereafter, the LCI was assessed, as reported in Table 9.

LCI analysis enables the aggregation of all materials, energy consumption, and gas emissions associated with the production of 20 kg of carbon materials from 100 kg of PET-PU post-industrial scrap within the system boundary. Moreover, it identifies the secondary outputs associated with the pyrolysis of PET-PU. It is possible to see that the two major outputs are the production of methane and carbon materials, as just found and analyzed in other research work [26,27,28]. The cradle-to-gate environmental performance for C-PET-PU was evaluated for the nine impact categories, and the results are presented in Table 10.

The outcomes of the study appear to substantiate the viability of pyrolysis as a means of transforming industrial textile waste into carbonaceous materials. With regard to global warming, the greatest contribution is made by the CO_2_ and CH_4_ emitted during the pyrolysis of PET and PU. With regard to the global warming potential (GWP), the impact is 1.41 kg CO_2_-eq/kg of C-PET-PU, which is lower than the value found in the literature [17]. In general, the impact categories for carbonaceous materials are more significant than those presented in this study. It is evident that the LCA results are contingent upon the quantity, nature, and credibility of the data gathered, the methodology employed, and the system boundaries delineated. The LCA conducted for the C-PET-PU is a preliminary study in comparison to the one conducted in the literature. Nevertheless, all the studies indicate that the most impactful process related to the production of carbonaceous material is the activation phase. Indeed, the activation process necessitates the utilization of diverse activation agents, including acids, bases, and organic compounds. In many instances, a second heating treatment is also required. For instance, Nowrouzi et al. conducted the LCA analysis on the preparation of activated carbons using H_3_PO_4_ and Cu(NO_3_)_2_·3H_2_O as activating agents. The LCA outcomes demonstrated that the release of H3PO4 contributed the greatest, with the copper ions also making a significant contribution. The impact of the activation process was also confirmed by a comparison among the carbons obtained from four different feedstocks (coal, peat, coconut, wood, and reactivated coal). The results indicate that the discrepancies in emissions between raw materials and their production methods are considerable and warrant further investigation. It is also crucial to obtain data on actual emissions during industrial production. In the case of C-PET-PU, no further activation is required for the production of microporous carbon materials with a high surface area and narrow porosity. The elimination of the activation phase has the effect of reducing the potential environmental impact of carbon production, thereby increasing the sustainability of the entire process. Nevertheless, further LCA analysis expanding the data collection and the system boundaries should be undertaken to corroborate these preliminary findings.

A preliminary LCC analysis was conducted to assess the economic viability and potential applicability of pyrolysis as a means of valorizing post-industrial PET-PU scraps. The cost estimation is presented in Table 11. All costs associated with the production are presented in accordance with the selected area (Italy) and the time period of 2024.

The total economic costs calculated are approximately 1.73 EUR/kg, in line with the results obtained in other research studies [17]. For instance, Nowrouzi and colleagues determined that the cost associated with the preparation of activated carbon is approximately between 1.5 and 1.9 EUR/kg. Adsorbents with the greatest potential (e.g., graphene, carbon molecular sieves, and microporous carbon materials) are more expensive to produce. As found by Heidari et al., the cost to produce high-performing carbon materials is approximately 250 EUR/kg [53]. In most cases, the cost of high-performing materials is related to that of the raw materials and activating agents. In this instance, the feedstock employed as a carbon precursor is a waste material. Consequently, apart from any potential costs associated with transportation, the cost is effectively zero. Moreover, no chemicals are employed during the formation of C-PET-PU, thereby further reducing the cost associated with the processing stage. Furthermore, the revenue associated with the potential sale of the C-PET-PU was evaluated. In this case, the price was established based on the physical and chemical characteristics of the product. The C-PET-PU can be classified as microporous carbon material, and its specific features were compared with those of commercial products. However, it is also important to consider pricing based on specific applications [53,54]. In the case of water remediation, for instance, it can be found that carbon materials, such as C-PET-PU, are typically sold for approximately 173 EUR/Kg. Based on this value, the revenue calculation reveals that the production of 20 kg of C-PET-PU results in a net profit of 2400 euros. Moreover, no chemicals are employed during the formation of C-PET-PU, thereby further reducing the cost associated with the processing stage. A more comprehensive cost analysis, which incorporates transportation, life-cycle, and regeneration costs, would undoubtedly provide greater precision in terms of revenue and processing costs.

### 3.12. Impact on Sustainable Development Goals

The results obtained regarding the production of porous carbon materials from PET-PU post-industrial scraps align with several United Nations Sustainable Development Goals (SDGs), particularly SDG 12: Responsible Consumption and Production and SDG 6: Clean Water and Sanitation. In particular, SDG 12 aims to ensure sustainable consumption and production patterns, reducing waste generation and promoting the efficient use of resources. The conversion of PET-PU waste into valuable microporous carbon materials directly contributes to this goal because the pyrolysis process transforms post-industrial PET-PU waste, which would otherwise be landfilled or incinerated, into high-value adsorbent materials. This upcycling approach extends the lifecycle of materials, minimizes waste, and conserves raw resources, aligning with the principles of the circular economy. By diverting waste from traditional disposal methods, this process reduces the environmental footprint associated with both waste management and the production of new materials. Moreover, simple pyrolysis eliminates the need for chemical activation, commonly required in traditional methods for producing activated carbon, thereby reducing the release of harmful chemicals and minimizing greenhouse gas emissions. Furthermore, the LCA results indicate that the global warming potential (GWP) of the pyrolysis process is significantly lower than conventional methods, contributing to a reduction in overall environmental impact.

As for SDG 6, it seeks to ensure the availability and sustainable management of water and sanitation for all. Indeed, the microporous carbon materials produced from PET-PU waste exhibit high adsorption capacities for common water pollutants, such as the two selected dyes. These materials provide an effective and sustainable alternative to traditional adsorbents for wastewater treatment, particularly in removing organic contaminants. The ability to regenerate and reuse these materials multiple times with minimal loss of adsorption capacity further enhances their suitability for continuous use in water purification systems. By providing an efficient method for removing contaminants from water, the adoption of these materials could improve access to clean water, particularly in regions with limited resources or high levels of water pollution. The low production costs and scalability of the pyrolysis process make it a feasible option for deployment in both urban and rural settings, potentially benefiting communities that rely on local water sources.

The production of microporous carbon for post-industrial textile waste can significantly contribute to achieving multiple SDGs by promoting sustainable production, improving water quality, and enhancing environmental resilience. By aligning research outcomes with global sustainability targets, this study underscores the potential of innovative material solutions to drive progress toward a more sustainable and equitable future.

### 3.13. Scale-Up Consideration

The transition from laboratory-scale synthesis to industrial-scale production of microporous carbon materials from PET-PU waste entails a number of pivotal considerations to guarantee the feasibility, cost-effectiveness, and sustainability of the process. These considerations encompass a range of factors, including technological, economic, and environmental considerations, which must be addressed in order to achieve a successful scale-up. The initial step is to select an appropriate reactor for the pyrolysis process. The scaling up of the pyrolysis process necessitates the selection and design of suitable reactors that are capable of handling substantial quantities of PET-PU waste while maintaining consistent temperature control, heat transfer, and inert atmosphere conditions. Furthermore, it is possible that some potential disadvantages may be associated with the pyrolysis temperature. Indeed, at an industrial scale, maintaining the desired pyrolysis temperature, typically around 800 °C, in a consistent and efficient manner becomes more challenging due to the increased thermal inertia of larger systems. The optimization of heat distribution, the reduction of heat loss, and the incorporation of energy recovery systems (e.g., recuperative burners, heat exchangers) are essential for the enhancement of energy efficiency and the reduction of operational costs. Furthermore, the addition of waste materials with higher calorific values could serve to supplement the energy required for the pyrolysis process.

From an economic perspective, the transition to an industrial scale entails a substantial capital investment in equipment, infrastructure, and plant facilities. The initial costs associated with acquiring and installing pyrolysis reactors, gas handling systems, heat exchangers, and material handling equipment must be justified by the anticipated production volumes and market demand for microporous carbon materials. It is essential to optimize operational costs, including energy consumption, labor, maintenance, and feedstock logistics, to ensure the economic viability of the process. Nevertheless, the scale-up process must be aligned with market demand for microporous carbon materials in a variety of applications, including wastewater treatment. An understanding of market dynamics, competitor analysis, and pricing strategies is essential for determining the optimal production scale and target prices, which are necessary for achieving a competitive position. Furthermore, potential collaborative arrangements with industries that generate PET-PU waste could provide a reliable feedstock supply while reducing raw material costs.

It is similarly crucial to take environmental compliance considerations into account. At the industrial level, emissions resulting from the pyrolysis process, including volatile organic compounds (VOCs), carbon monoxide (CO), methane (CH_4_), and other pollutants, must be meticulously managed to ensure compliance with local and international environmental regulations. The implementation of appropriate emission control technologies, such as scrubbers, filters, and catalytic converters, is crucial for the reduction of environmental impact. Furthermore, the implementation of continuous monitoring systems may be necessary to guarantee compliance with established air quality standards. Consequently, a comprehensive LCA is required to evaluate the environmental impact of the scaled-up process, from feedstock acquisition to end-of-life disposal. This analysis will assist in the identification of potential areas for the reduction of the carbon footprint, energy consumption, and waste generation. In order to enhance the overall sustainability of the production process, it would be prudent to explore strategies such as the use of renewable energy sources, optimization of logistics, and recycling of by-products. Furthermore, it is essential to consider the social and ethical implications of scaling up the production process, including the potential for job creation in the recycling and manufacturing sectors, the health and safety of workers, and the broader community impact. It is therefore vital to ensure fair labor practices, promote safe working conditions, and engage with local communities in order to achieve social acceptance and support.

### 3.14. Comparative Analysis of C-PET-PU with Other Microporous Carbon Materials

Microporous carbons are widely used in various applications, including wastewater treatment, gas storage, and energy storage, due to their high surface area and tunable porosity. This section compares the structural, adsorption, and environmental characteristics of C-PET-PU with other microporous carbons derived from different feedstocks. The comparison aims to elucidate the advantages and potential limitations of C-PET-PU as an adsorbent material. In order to perform this comparison, the following materials have been taken into consideration:

1. C-PET-PU is derived from post-industrial PET-PU waste through pyrolysis.
2. Activated carbon is a highly porous form of carbon with a large surface area. It is produced from carbonaceous materials through processes like thermal or chemical activation and used primarily for adsorption to remove contaminants from gases or liquids.
3. Biochar is a charcoal-like material produced through the pyrolysis of biomass. It is obtained from agricultural waste, such as rice husks, through pyrolysis.
4. Graphene oxide (GO) is a two-dimensional carbon material that exhibits unique chemical and physical properties. The synthesis of GO from graphite is achieved through the application of the Hummers method.

The four types of carbon were compared based on three criteria: specific surface area, environmental sustainability, and economic feasibility.

With regard to the specific surface area, Table 12 presents a comparative overview of the range of values documented in the literature for the three carbon types under consideration, along with the C-PET-PU data.

The specific surface area of biochar is typically low, and the pore width is relatively large, which limits its potential for use in adsorption applications. By contrast, graphene oxide is a carbon material with an extraordinary surface and an intriguing porosity. These properties render it a versatile material. The C-PET-PU exhibits a specific surface area that falls between that of biochar and the typical range for activated carbons. Despite this intermediate range, the C-PET-PU is well suited for specific adsorption applications.

With regard to environmental sustainability, Table 13 provides a summary of the data for the three types of carbon analyzed, as well as the data obtained for C-PET-PU.

It is evident that biochar consistently presents the most significant environmental advantages, particularly in terms of carbon sequestration and the utilization of renewable feedstocks. Nevertheless, the C-PET-PU represents a sustainable alternative, as it employs waste as a feedstock and avoids the use of harmful activation chemicals, thereby minimizing its environmental impact.

Although a material may be environmentally sustainable, it is also important to know its economic feasibility. For this reason, the economic feasibility of the carbon materials under consideration is shown in Table 14.

It can be reasonably deduced that GO is the most expensive option. The production costs associated with GO are considerable, which limits its use to high-value applications. It can be reasonably asserted that biochar is the most cost-effective material, given its ease of production and the potential for lower market prices due to its limited applications. It is for this reason that the most successful carbons are activated. From a chemical and physical standpoint, active carbon boasts a plethora of exceptional characteristics, rendering it an ideal candidate for utilization in a multitude of applications. Nevertheless, the necessity for an activation process renders this type of coal less environmentally sustainable than economically viable. Accordingly, a viable compromise between active carbon and biochar could be C-PET-PU. Indeed, C-PET-PU presents a competitive production cost, with the potential for profit due to the low cost of the feedstock and the simplicity of the processing.

## 4. Conclusions

This study demonstrates the feasibility and benefits of converting polyester–polyurethane (PET-PU) post-industrial waste into high-performance microporous carbon materials for environmental applications. Through a straightforward pyrolysis process, PET-PU waste was successfully transformed into a carbon adsorbent with a high specific surface area (632 m^2^/g) and narrow porosity range (2–10 Å), effectively removing methylene blue and orange II dyes from aqueous solutions.

The adsorption experiments revealed that C-PET-PU exhibited a high adsorption capacity for methylene blue, following a pseudo-second-order kinetic model, which suggests a strong interaction between the adsorbent and dye molecules. The regeneration and reusability studies further demonstrated that C-PET-PU retained over 85% of its original adsorption capacity after five cycles, underscoring its potential for sustainable and cost-effective wastewater treatment.

The life cycle assessment (LCA) and life cycle cost (LCC) analysis highlighted the environmental and economic advantages of using PET-PU waste as a raw material. The process significantly reduces the global warming potential and production costs compared to conventional activated carbons, aligning with the principles of the circular economy and sustainable resource management.

Compared to other microporous carbon materials, such as activated carbon, biochar, and graphene oxide, C-PET-PU offers a balanced combination of sustainability, cost-effectiveness, and adsorption capacity, making it a competitive alternative for various applications.

Future research should focus on optimizing the pyrolysis process to further enhance the structural properties of C-PET-PU and exploring its adsorption capabilities for a wider range of pollutants. Additionally, scaling up production and conducting pilot-scale experiments will be crucial for validating its commercial potential and expanding its application scope in industrial wastewater treatment.

In summary, the conversion of PET-PU waste into valuable carbon materials presents a promising solution to the challenges of textile waste management and environmental remediation, contributing to sustainable development and the reduction of environmental impacts.

## Figures and Tables

**Table 1:** Activation energies for PET-PU pyrolysis using KAS and FWO methods.

Degradation Stage	KAS Ea (kJ/mol)	FWO Ea (kJ/mol)
PU degradation	150 ± 5	152 ± 4
PET degradation	240 ± 10	238 ± 9

**Table 2:** Comparison between the simulated and the experimental characteristics of the C-PET-PU.

	Theoretical Data	Experimental Data
**Specific surface area**	635 m^2^/g	632 m^2^/g
**Pore volume**	0.80 cm^3^/g	0.80 cm^3^/g
**Pore width**	2–10 Å	2–10 Å

**Table 3:** Dye absorbed by C-PET-PU at different dye concentrations (RT).

Initial Dye Concentration (ppm)	MB Adsorbed (ppm)	OII Adsorbed (ppm)
10	5.80	3.43
50	42.20	22.70
100	64.70	28.18
150	66.90	28.30

**Table 4:** Thermodynamic parameters related to the adsorption of OII and MB onto C-PET-PU.

*OII adsorption*					
Initial Dye Concentration (ppm)	ΔH^0^ (j/mol)	ΔS^0^ (j/molK)	ΔG^0^ (j/mol)		
			**303 K**	**323 K**	**343 K**
1	−83.31	0.055	−99.98	−101.08	−102.18
3	−89.67	0.073	−111.79	−113.25	−114.71
10	−106.74	0.15	−152.19	−155.19	−203.64
***MB adsorption***					
**Initial dye concentration** **(ppm)**	**ΔH^0^** **(j/mol)**	**ΔS^0^** **(j/molK)**	**ΔG^0^** **(j/mol)**		
			**303 K**	**313 K**	**343 K**
1	−342.83	0.88	−609.47	−618.27	−644.67
3	−309.82	0.86	−567.37	−575.87	−601.37
10	−140.63	0.38	−255.77	−259.57	−270.97

**Table 5:** Kinetic parameters for the adsorption of direct red 7 on virgin and recycled cashmere.

Sample	Pseudo-First-Order		Pseudo-Second-Order		
	R^2^	k_1_ (min^−1^)	R^2^	q_e_ (mg/g)	k_2_ (g/mg min)
OII	0.782	−0.001	0.999	0.55	0.286
MB	0.753	3 × 10^−4^	0.997	6.90	0.154

**Table 6:** C-PET-PU MB adsorption in comparison to other adsorbents.

Adsorbent Material	Dye	Adsorption Capacity (mg/g)	Preparation Method	Ref.
C-PET-PU	MB	169.49	Pyrolysis (PET-PU blend)	
Sunflower oil cake	MB	15.80	Chemical activation	[47]
Bituminous coal	MB	588	Physical activation	[48]
Citrus fruit peel	MB	25.51	Chemical activation	[49]
Waste PET bottle	MB	404.09	Chemical activation	[50]
PET	MB	838.012	Chemical activation	[51]
Bamboo	MB	454.2	Physical activation	[52]

**Table 7:** Regeneration efficiency of C-PET-PU after five cycles.

Cycle	Adsortion Capacity (mg/g)	RE (%)
1	169.49	100
2	162.32	95.8
3	156.89	92.5
4	151.23	89.2
5	147.56	87.0

**Table 8:** Major constituent of the gaseous products of PET and PU.

Constituent of Gaseous Products	PET (%)	PU (%)
Carbon dioxide	1.27	0.33
CH_4_	19.54	1.4
H_2_	9.57	/
CO	4.45	/
Toluene	4.00	4.7
Benzene	/	4.6

**Table 9:** Life cycle inventory of PET-PU conversion into carbon material.

Input			Output		
*Item*	*Quantity*	*Unit*	*Item*	*Quantity*	*Unit*
PET	87.00	Kg	Carbon	20.00	Kg
PU	13.00	Kg	Nitrogen	25.70	g
Nitrogen	25.70	g	CH_4_	16.89	Kg
Energy consumption	560	KJ	CO_2_	1.50	Kg
			Toluene	4.30	Kg
			Other gases	57.31	Kg

**Table 10:** Environmental impact and contribution of parameters associated with the pyrolysis scenario.

Impact Category	Value	Unit
Ozone depletion	/	Kg CFC-11-eq
Climate change	4.8	Kg CO_2eq_
Particulate matter formation	/	Kg PM_2.5-eq_
Eutrophication	/	Kg N-eq
Human cancerogenics	2.06 × 10^−14^	CTUh
Human non-cancerogenics	5.81 × 10^−7^	CTUh
Ecotoxicity	14.04	CTUe
Photochemical oxidant formation	4.50 × 10^−3^	Kg C_2_H_4_-eq
Acidification	/	Kg SO_2_-eq

**Table 11:** Cost analysis for the preparation of 20 Kg of C-PET-PU.

Item	Quantity	Unit Cost	Total Costs
Nitrogen	25.70 g	0.60 EUR/g	15.42 EUR
PET-PU	100 Kg	0 EUR/Kg	0 EUR
Energy consumption	560 Kj	0.010 EUR/Kj	5.60 EUR
Emission (CO_2_)	1.50 Kg	2.40 EUR/Kg	3.60 EUR
Emission (TOT)	16.89 Kg	0.60 EUR/Kg	10.13 EUR
**TOTAL**			34.75 EUR

**Table 12:** Specific surface area and porosity of the considered carbon materials.

Materials	Specific Surface Area (m^2^/g)	Pore Volume (cm^3^/g)	Average Pore Size (Å)	References
C-PET-PU	632	0.8	2–10	/
Activated carbon	700–1800	0.39–0.923	5–15	[55]
Biochar	78–550	0.009–0.052	10–20	[56]
GO	2300–2400	0.11–1.2	<2	[57,58]

**Table 13:** Sustainability and environmental impact of the considered carbon materials.

Material	Feedstock	Environmental Impact	Renewability	References
C-PET-PU	PET-PU waste	Low GWP, no activation	High	/
Activated carbon	Coconut shells	Moderate GWP, chemical use	Moderate	[59]
Biochar	Agriculture waste	Low GWP, carbon sequestration	High	[60]
GO	Graphite	High GWP, chemical use	Low	[61]

**Table 14:** Cost associated with the production of the carbon materials.

Materials	Production Cost (EUR/kg)	Market Price (EUR/kg)	References
C-PET-PU	1.65	3	/
Activated carbon	2.5	5	[62]
Biochar	1.2	2	[63]
GO	50	100	[64]

## Data Availability

Data are contained within the article.
